# Pain: metaphor, body, and culture in Anglo-American societies between the eighteenth and twentieth centuries

**DOI:** 10.1080/13642529.2014.893660

**Published:** 2014-03-20

**Authors:** Joanna Bourke

**Affiliations:** ^a^ Department of History, Classics, and Archaeology, Birkbeck, University of London, London, UK

**Keywords:** pain, metaphor, body, culture, history, British, American, cognitive linguistics

## Abstract

This article explores the relationship between metaphorical languages, body, and culture, and suggests that such an analysis can reveal a great deal about the meaning and experience of pain in Anglo-American societies between the eighteenth and twentieth centuries. It uses concepts within embodied cognition to speculate on how historians can write a history of sensation. Bodies are actively engaged in the linguistic processes and social interactions that constitute painful sensations. Language is engaged in a dialogue with physiological bodies and social environments. And culture collaborates in the creation of physiological bodies and metaphorical systems.

## 1. Introduction

Pain undermines mind–body dichotomies: the cry ‘it hurts, *here*!’ is both an assertion about the localization of pain in the body and a testimony to amorphous suffering. This article explores those languages that people seize hold of in order to overcome some of the obstacles they face when attempting to express pain. Figurative languages are central to all attempts at communicating unpleasant sensations to oneself as well as to others. Pain-talk is swollen with metaphor, simile, metonym, and analogy. Why are such linguistic devices so crucial to painful experiences? Can the exploration of the figurative or metaphorical languages of pain enable us to speculate on the historical changes in the sensation of pain? I will be arguing that an analysis of the dynamic interconnections between language, culture, and the body can contribute to a history of sensation.

The three concepts of language, culture, and the body are shorthand terms for complex phenomena. None are discrete entities: each exists in relationship to the others, and an adjustment in one inevitably modifies the other two. First, my approach to ‘language’ is influenced by the non-representational theories of phenomenological philosophers, in which language is intricately intertwined with ‘lived experience’. This approach has its critics, some of whom argue that a historical study of pain (to take the example that interests me here) is nothing more than a study of *representations* of pain. In other words, language is ‘“about” nothing other than itself’ or, in another version, it ‘wholly constitutes experiences’. Social theorist Thomas Csordas has a nice retort to this critique, pointing out that the ‘polarization of language and experience is itself a function of a predominantly representational theory of language’. Instead, he argues, it is perfectly plausible to argue that language:

gives access to a world of experience in so far as experiences comes to, or is brought to, language … The notion that language is itself a modality of being-in-the-world … is perhaps best captured in Heidegger's notion that language not only represents and refers, but ‘discloses’ our being-in-the-world. (Csordas 1994, 11)

Metaphor plays a central role in these acts of disclosure, particularly in the context of pain-talk. For this reason, most of my discussion of language refers to the full range of figurative languages.

Second, the term ‘culture’ is shorthand for social and environmental interactions. It includes both interpersonal relations as well as people's engagement with all aspects of their surroundings. Finally, when I refer to ‘the body’ I am not referring to a natural, pre-social entity. Although there are material components to the body, the concept ‘body’ is not interchangeable with physiology. The body is always more than a sum of its corporal parts. At times, I will refer to the ‘physiological body’, but this is simply a rhetorical stratagem for drawing attention to the fact that bodies are also fleshy, fluid entities, but nevertheless accessed and experienced through language.

In this article, therefore, I seek to show how these three strands are inextricably entangled in the context of figurative languages generally, and pain-languages specifically in Anglo-American cultures from the eighteenth century onwards. As we shall see, bodies are not simply receptacles of sensations, but are actively engaged in the linguistic processes and social interactions that constitute those sensations. Language is engaged in a dialogue with physiological bodies and social environments and culture does not simply ‘inscribe’ its texts upon a natural, pre-social body, but collaborates in the creation of physiological bodies and metaphoric systems. In this way, this ‘language, culture, body’ model is an interactive one, each strand intertwined in an active, dynamic relationship.

## 2. The importance of metaphorical languages

It may be useful to say a few very general words about metaphor, before I go on to a more detailed analysis of the relationship between metaphorical languages, body, and culture. I will be using the term metaphor in its broadest sense, that is, as a rhetorical figure of speech that employs association, comparison, or resemblance. In this way, metaphor refers not only to an analogy between two things (‘pain gnawed at his stomach’), but also as a simile (‘the pain felt like a rat, gnawing his stomach’) and a metonym (‘the gnawing continued’).

Etymologically, metaphor comes from the Greek words *meta* and *pherin*, or ‘to transfer’ and ‘to carry beyond’. Through metaphor, a concept is transferred into a context within which it is not usually found, extending its meaning. Metaphors enable people to move a subject (in this case, pain) from inchoateness to concreteness. As such, metaphor is not simply an ornament of communication but, as cognitive scientist Raymond Gibbs observed, it is a ‘specific mental mapping that influences a good deal of how people think, reason, and imagine in everyday life’ (1999, 145). Human language is thoroughly infused with metaphoric figures of speech. Indeed, they cannot be avoided. Thus, Sontag's (1990) celebrated assertion in *Illness as Metaphor* that metaphors are inherently stigmatizing and must be avoided in illness narratives is not only wrong, but also impossible. Ironically, her book is brimming with opulent and elegant metaphors (1990).

Metaphors are particularly useful when people are attempting to convey experiences most resistant to expression. Indeed, because pain-narratives are most often fragmentary, rather than elaborate accounts, the analysis of metaphors can be particularly rewarding for historians of pain. It is difficult to imagine how people could communicate (to themselves as well as to others) the sensation and meaning of pain without metaphoric crutches. Crucially, by using metaphors to bring interior sensations into a knowable, external world, sufferers attempt to impose (and communicate) some kind of order onto their experiences.

## 3. The body and the creation of metaphor

Figurative languages have an even greater significance than this implies. It is useful to turn to that remarkable book entitled *Metaphors We Live By (1980)*, in which linguist George Lakoff and philosopher Mark L. Johnson argue that metaphors are based on embodied experiences. Crucially, these intrinsically embodied metaphors are integral to cognitive processes. Metaphor ‘is not just a matter of language, that is, of mere words’, they observed. Rather, ‘human *thought processes* are largely metaphorical’ (1980, 6). Linguists and philosophers have subsequently developed this relationship between metaphor and the body, and, I will be arguing, their insights can be used to underpin a history of sensation.

A crucial starting point is the inherent embodiment of consciousness. As philosopher Maurice Merleau-Ponty argued, we do not *own* our bodies; we *are* them (Merleau-Ponty [1945] 2002, np [Part I]). Furthermore, the subjective character of experience (its phenomenological content) does not simply arise from interactions in the world but is constituted by those interactions. In *The Body in the Mind* (1990), Johnson observed that reality is ‘shaped by the patterns of our bodily movement, the contours of our spatial and temporal orientation, and the forms of our interaction with objects’ (1990, xix). This view is expressed even more concisely in Lakoff and Johnson's *Philosophy in the Flesh* (1999). ‘Our mind’, they insist, ‘is embodied in the profound sense that the very structure of our thoughts come from the nature of our body’ (6). Gibbs was also drawing upon the dialectic between body and language when he contended that people's ‘embodied experiences give rise to their metaphorical structuring of abstract concepts, which in turn, constrains speakers’ use and understanding of language' (1999, 148). Basic bodily actions, such as ‘pushing, pulling, grasping, standing, walking, and interacting with a physical environment’ provide the more ‘elementary forms of knowledge’, psychiatrist Laurence Kirmayer explained, while ‘more abstract concepts are built on a scaffolding of simpler metaphors which in turn can be traced back to sensorimotor image schemas’. In this way, metaphors ‘bridge the bodily given and the culturally configured social world’ (2007, 369, 371).

In order to understand how they came to such conclusions, a detailed exposition of certain concepts is necessary. It will be followed by a discussion about the ways that historians can use such concepts in *non-reductionist* way in order to illuminate changes over time in pain-languages. Language, body, and culture turn out to be inextricably entwined.

According to cognitive linguists, people form image schemata out of sensorimotor bodily experiences, which are then projected metaphorically onto the wider world. These image schemata (generally written in small-caps, as in balance or containment) are dynamic gestalt patterns that are based on recurring features in the physical interaction between the environment and the body. Take, for example, the image schema balance. Balancing, as Johnson explains, depends on bodily experiences. It ‘is an *activity we learn with our bodies* and not by grasping a set of rules or concepts’ (1990, 74). It is related to ‘equilibrium in the body, whereby we try to maintain an even state – for example, with respect to heat or cold’ (Strathern 1996, 183). balance therefore forms an image schemata that can then be projected on (or elaborated in) other experiences: as in ‘pain weighed down her spirits’. Similarly, the containment schema starts from the relationship between the body's boundaries and its interiority/exteriority, and is used metaphorically in statements such as ‘she felt pressure on her spleen’. The sensorimotor bodily experience of lying down to sleep and standing up when awake is the corporeal basis for the metaphor happy is up and sad is down (in the sense that ‘his spirits rose’ or ‘she fell into a depression’). Similarly, the metaphor that health/life are up and illness/death are down (as in ‘He suddenly felt buoyant’ or ‘He was sinking fast’) are based on physical comportment relating to vigour versus infirmity (Lakoff and Johnson 1980, 15). In each of these instances, the ‘source domain of the metaphor comes from the body's sensorimotor system’ (Gibbs 2006, 99).

The argument that ‘metaphor is in the body’ can be illustrated further by looking at two conceptual metaphors: intensity is heat and anger is heated fluid in a container. Linguist Zoltán Kövecses asks readers to imagine that they are engaged in hard labour:

After a while you are beginning to work up heat, you feel hot, and maybe you begin to sweat. We can say that the vigorous bodily activity produces an increase in bodily heat … Similarly, when you are very angry, or when you have strong sexual feelings, or when you are under strong psychological pressure, your body may also produce an increase in bodily heat that manifests itself physiologically in a variety of ways. In all of these cases, the increase in the intensity of an activity or state accompanies an increase in body heat, or your body responds this way automatically.

This bodily correlation forms the basis of the conceptual metaphor intensity is heat: the correlation between intensity and heat occurs ‘at the level of the body; and in this sense metaphor is as much in the body as it is in language or thought’ (Kövecses 2005, 18).

Similarly, the schema container is behind the conceptual metaphor anger is heated fluid in a container (examples would include ‘he blew his stack’, ‘she hit the roof’, and ‘it made her blood boil’). People, Gibbs explains:

have strong kinaesthetic experiences of bodily containment ranging from situations in which bodies are in and out of containers (e.g. bathtubs, beds, rooms, houses) to experiences of bodies as containers in which substances enter and exit. An important part of bodily containment is the experience of our bodies being filled with liquids including stomach fluids, blood, and sweat. Under stress, people experience the feeling of their bodily fluids becoming heated. These various, recurring bodily experiences give rise to the development of an experiential gestalt, called an *image schema* for containment. (1999, 148)

These image schemas are used metaphorically to help people understand and communicate more abstract concepts. For instance, the containment schema is ‘metaphorically elaborated to explain some of the complex ways that we structure single abstract concepts’, such as anger. Again, in the conceptual metaphor anger is heated fluid in a container, people:

know that when the intensity of anger increases, the fluid in the container rises (i.e. ‘his pent-up anger welled up inside him’), people know that intense heat produces steam and creates pressure on the container (getting hot under the collar – blowing off steam – bursting with anger), and people know that when the pressure of the container becomes too high, the container explodes (‘She blew up at me’). (Gibbs 1999, 148)

In these examples, body-based schemata are transferred though metaphor from one (bodily) context to another.

## 4. Pain-metaphors and the body

Given the ways that painful sensations affect autonomic arousal, cardiovascular responses, and sensorimotor actions, it is not surprising that body-based schemata are central to languages of pain. For instance, the most common conceptual metaphor in pain-speech is physical states are independent entities. For example, pain was a ‘monster’ (Anon. 1898, 38). In her First-World-War diary, a member of the Voluntary Aid Detachment (VAD) recalled a patient who would wake up sobbing with pain: ‘Don’ [*sic*] go away nurse', he would beg, and then he would point into the rafters, cry ‘There's the pain!’ (Bagnold 1918, 102). As an independent entity, pain could be all-enveloping. Mrs M. was a terminally ill patient in St. Joseph's Hospice in 1961. She conceived of her pain as an independent entity that so entirely enclosed her body that even the *approach* of other people aroused it. In her words:

It – I would say the pain was so bad that I dreaded anyone touching me and when anyone knocked my bed or came near me – the first thing I said to them – ‘please don't touch me. Please don't move me’ … It was an obsession in a way because it was all round me.

Powerful analgesics were experienced as cushioning her from this oppressive, independent entity. Pain-relief made her feel:

very comfortable indeed. It it [*sic*] seemed to be that … there was something between me and the pain. It was like a nice thing wrapping around me. It was. (‘Mrs. E’, ‘Conversations with Patients, Recorded During Research Conducted at St. Joseph's Hospice, Hackney, East London, During a Sir Halley Stewart Trust Fellowship’, 1963, np)

Conceiving of pain as a separate entity could, in fact, help exert power over that unpleasant entity. As Nietzsche famously quipped, ‘I have given a name to my pain’: it is called “dog”'. In this way, Nietzsche's pain was ‘just as faithful, just as obtrusive and shameless, just as entertaining, just as clever as any other dog, and I can scold it and vent my bad mood on it, as others do with their dogs, servants, and wives’ (1974, 249–250).

An extended version of this conceptual metaphor was physical states are independent entities *within* a person. In the words of a wagon driver, pain ‘throbs and darts as if something was running though it’ (Wesbrook 1778–81, np). In 1901, Alice A. complained of a pain in her head: she felt as though ‘something about an inch long were moving about in her throat, and as though the top of her head were being pricked and being moved up and down’ (Bulstrode 1901, 4). Humourously, *The [Adelaide] Advertiser* used this metaphor in a sketch entitled ‘The Pain’ (Anon. 1927), in which an ‘Anxious Mother’ asks: ‘You don't look well, Johnny. Are you in pain? Johnny responded with ‘No, mummy. The pain's in me’ (Anon. 1927, 8).

The conceptual metaphor intensity is heat is another common one. Pain was heat: it was fire or sun; it seared, boiled, burnt. It was a ‘spark of fire, shooting up the wounded finger’ (Anon. 1836, 27), ‘like a hot iron being placed on his right ear’ (Anon. 1899a, 7); and a ‘terrible sensation of molten lead running down my arm’ (Leriche 1939, 65). In the 1860s, labourer Joseph Townend described his wound (caused by undergoing an excruciating operation without anaesthetics) as ‘smoking’. Later, when the only partially healed lesion was violently torn open again, he observed that ‘my poor side was drenched in blood, and smoked, almost like a kiln’ (1869, 12–13, 18). This was also the metaphor that came most readily to the pen of Anthony Babington. When reflecting on the suffering caused by his war wounds and tuberculosis, Babington claimed that it was like ‘walking for a long time in the sweltering heat of a fierce sun’; death would be ‘sweet and merciful – like the shade of a great tree’ (1954, 184).

The third important conceptual metaphor – the body is a container – is not surprising, given that wounds and illness often do represent a fracturing of bodily integrity. Often, an agent was identified. It was a knife: cancer pains were ‘like that of a stab of a knife’ (Ure 1852, 735) or, as another woman complained a few years later, ‘It seems, at each breath, as if a knife were passing through me … It seems as if a heavy weight were crushing in my breast’ (Arthur 1858, 183). Pain was ‘like knives’, patients grumbled in the 1870s: it was a ‘knife-like spasm’, or ‘as though someone was giving … repeated violent digs with a knife’ (Buzzard 1878, 168, 172–173). In 1895, a 44-year-old fireman at the Royal Infirmary described his pain in his shoulder as ‘dead burning heat … like red-hot sand or iron passing through the flesh … A knife driving straight in … Shooting … cutting’ (Monro 1895, 567). A patient, in 1950, claimed that pain was ‘like a knife stuck to the bone’ (Hubbell 1950, 204).

Other times, hammers were identified as the weapon breaching the integrity of the body. In 1894, a working-class woman described how ‘Sometimes I feel like a hammer knocking in my belly. This week my breasts bite me so, that I cannot describe it’ (‘Letter from Mrs. S’ 1894, 52). For Mrs C., speaking from St. Joseph's Hospice in Hackney in 1962, pain was both a hammer and a crushing vice. It was:

just as bad as it could be. I couldn't breath … in, and I couldn't breath … out, could bring nothing up, could force nothing down … it was just as if I was in a vice, being crushed … The chief trouble … was the pain behind the shoulder blade … it used to throb as if someone were bumping into it with a big hammer. (‘Mrs. C.’ 1962, np)

Other weapons were identified. In the early 1900s, sinus pain felt like a ‘red-hot circular saw’ (Gray 1938, 90). Or, a constraining rope was responsible for attacking the body's integrity. In 1811, a ‘violent fixed pain at the pit of the Stomach’ was described as if it were ‘bound round with a cord’ (Rees 1811, 151).

In each of these examples, the body is not simply the container for feeling and acting, but a way of thinking as well. In such ways, autonomic arousal, cardiovascular responses, and sensorimotor actions influence the way people think: the body provides possibilities (including constraints) for the metaphors adopted. An analysis of such conceptual metaphors illustrates some of the ways in which people *think* via sensorimotor experiences: our minds are embodied. In Gibbs’ evocative phrase, ‘cognition is what happens when the body meets the world’ (1999, 153).

## 5. Culture creates metaphor

These ways of thinking about metaphor and the body are useful, but they come up against an important problem. Does not the model threaten to ‘flatten out’ pain-descriptions and universalize the body? Is not the physiological body the same everywhere? If so, should not metaphors be remarkably similar all over the world? Linguist Ning Yu believes the answer to these two questions is ‘yes’. Despite ‘racial or ethnical peculiarities’, she notes, people ‘all have the same basic body structure, and all share some common bodily experiences and functions, which fundamentally define us as being human’. As a consequence, she reasons, it ‘also follows that our body … is a potentially universal source domain for metaphorical mappings’ (2008, 389). In other words, if metaphors are drawn from physiological sensations, then they must be transhistorical and transnational.

In *Metaphor in Culture* (2005), Kövecses grapples with this issue. He notes that the standard view of cognitive linguists is that metaphors are non-problematically grounded in embodied experiences. Thus:

We metaphorically view affection as warmth … because of the correlation in our childhood experiences between the loving embrace of our parents and the comforting bodily warmth that accompanies it. This gives us the ‘conceptual metaphor’ affection is warmth … Thinking (by means of affection is warmth) and talking (e.g. We have a *warm* relationship) of affection in terms of warmth arises naturally from our embodied experience. Probably no one would be surprised to hear that affection is universally conceptualised as warmth, rather than coldness. To learn such ‘primary’ metaphors is not a choice for us: It happens unconsciously and automatically. Because this is a universal bodily experience, the metaphor corresponding to it may well be universal. (2005, 2–3)

Superficially, it sounds plausible. But Kövecses goes on to identify an obvious problem: even a cursory look at the world's languages reveals a formidable number of non-universal metaphors. He does not use pain as an example, but metaphorical diversity is a key feature in pain-speech. For instance, Latinos in North America distinguish between a headache (‘dolor de cabeza’) and a brain-ache (‘dolor del cerebro’) (Abad and Boyce 1979, 34). The McGill Pain Questionnaire (an extensive list of pain-descriptors that was developed in America in the 1960s) could not always be translated straightforwardly into other European languages. As two Finnish experts reported:

It is not possible to translate this kind of specialized vocabulary into other languages without losing its validity, since no dictionary contains reliable and meaningful category/intensity equivalents. (Pöntinen and Ketovuori 1983, 85)

Indeed, they discovered, the ‘punishment’ category of the questionnaire, with its English-language connection to the idea of retribution for some real or imagined sin, was simply incomprehensible to Finnish speakers. ‘Is it that the Finnish cultural milieu is unable to associate pain with punishment or merely that the words given just did not connect with the emotions characterized by it?’, they wondered (Ketovuori and Pöntinen 1981, 252).

When turning to pain-terms in Asia and India, the differences multiply. While in English it is common to say ‘I have a pain’, implying that the sufferer possesses an object or entity, this is not the case in Thai, where the language of pain is much more active and dynamic. As Fabrega and Tyma explained:

the absence of nominal primary pain terms in Thai means that it is more difficult to qualify pain directly through metaphor as is done in English … In English, the process of metaphorisation allows the speaker to qualify his experience in a vivid and direct manner: I have a burning pain, I have a firing pain, etc … and his overt behavior often reflects this qualification. The native Thai is not provided with this flexible device of metaphorisation in describing his pain … Pain descriptions in Thai are somewhat ambiguous and it would appear that for semantic focus speakers are dependent on context. (1976, 329–330, 332)

The Sakhalin Ainu of Japan complain of ‘bear headaches’ that resemble the heavy steps of a bear; ‘musk deer headaches’, like the lighter galloping of running deer; and ‘woodpecker headaches’, as if pounding into the bark of a tree. Crucially, chills are not present during these kinds of headaches. Headaches that presented themselves with a chill required aquatic animal metaphors: such as an ‘octopus headache’ with its sucking motion or a ‘crab headache’ with its distinctive, prickling sensation (Ohnuki-Tierney 1981, 49–50). In China, pain-narratives are strongly affected by the traditional Chinese medical ideas about imbalance. Thus, metaphors for headaches revolve around notions of vertigo or painful dizziness (Ots 1990, 34). Chinese metaphors are also much more likely than English ones to refer to body parts, also explicable in terms of the theories of yin–yang and the five elements of Chinese medicine (Yu 2009). Cambodian:

distinguishes a type of internal tugging, throbbing or cramping pain … Sinhala distinguishes pain thought to be associated with an ‘ill wind’ (‘emma’), which can affect the head, back, etc.; a different type (‘rudava’) affects the eyes, ears, teeth and throat. (Diller 1980, 22)

In India, pain's hotness is imaged not only as fire and live coals, but also as ‘parched chickpeas’ and its heaviness is compared with ‘a load of grain’. Like many other countries, in India, everyday languages of pain do not distinguish between bodily discomfort and emotional suffering (Pugh 2005, 117–118). As Fabrega and Tyma observed after analysing pain-languages in English, Thai, and Japanese, ‘to the extent that culture and language may actually affect perception, thought and cognition, then to that extent they may also affect the actual experience of pain’ (1976, 332).

## 6. Culture and the creation of physiology

Later in the article, I will be addressing the issue of non-universal metaphors for pain in greater detail, developing the argument that it is insufficient to focus on only two of the elements (that is, language and the individual's body) in my three-stranded model. Metaphor-creation arises out of the body, but it is also a social phenomenon: we need to interrogate further the third of my strands – that of culture.

But before I expand upon that argument, it is important to note that there is another way to respond to the question of why a universal human physiology does not lead to universal metaphors: that is, to question what we mean by physiology. This is not the same as arguing that different cultures or people in different periods of history have *evaluated* physiology in distinctive ways. Ning Yu, for instance, admits that culture has ‘an interpretative function in viewing the body and its role in grounding metaphor’. Identical parts of the body or physiological processes could have differing significance for distinctive groups of people. Consequently, she states, it is not surprising that ‘in different cultures and languages, different body parts or bodily experiences are selected to map onto and structure the same abstract concepts’ (2008, 393).

Of course I agree with Yu (and will say more about these selective processes later), but her argument does not go far enough. In her model, what is important is the way different cultures *interpret* or *value* bodily parts and processes. These evaluative differences certainly exist and have a major role to play in explaining different metaphorical mappings. But, for Yu, human physiology itself remains a given. This is where we disagree. I will be arguing that physiology is profoundly affected by culture and metaphor.

First, no physiologist will disagree with the statement that individuals possess subtly different physiologies. Many physiological facts are about probabilities. Muscles that are not used, atrophy, and neurological faculties that are ‘exercised’ develop in different ways to those that are ignored. Individual physiologies are each unique, having been affected by distinctive DNA and molecular structures, feedback systems, conditioned reflexes, and so on. In Anglo-American societies, the so-called universal human body has generally been predicated upon the male exemplar and a *particular* positioning of bone, tissue, muscle, fluids, and fat. However, human physiology is much more diverse in shape and function (fe/male; dis/abled; petite/obese) than posited by this model. Not everybody is physiologically capable of menstruation, nocturnal emissions, labour pains, lactation, or beard-growing, to take just a few examples. Different bodies have different physiologies and they therefore *feel* different. We would expect to see metaphors reflecting these differences.

Second, it is worth asking: what is meant by ‘physiology’. No one is doubting that the human body is a material object, made up of fluids, fat, tissue, muscle, and bone, all encased in skin and embellished in practical ways with hair and nails. No matter who you are, your blood ‘circulates’, your nerves ‘fire’, your neurons ‘light up’. But these ways of understanding the ‘facts’ of physiology are based on metaphor. It is not enough to say: abolish the metaphor, and blood will still circulate, nerves will still respond sympathetically, and neurons will continue to transmit signals. The point is that the very way in which people and cultures metaphorically fashion physiology has profound effects on what that physiology *is*. The personal body, Haraway correctly argues, is not ‘natural, in the sense of existing outside the self-creating process called human labour’ (1991, 10). The physiological body is not a culture-free object. At every point, the facts of physiology are given cultural meanings and these meanings are not something that exist in a pre-social universe, but are an integral part of the very organization of that physiology. In other words, it is not simply the case that culture ‘inscribes’ something on a ‘natural’, pre-social physiology, but that physiological processes cannot be separated from the various and varying cultural meanings given to fluids, fat, tissue, muscle, bone, hair, and skin. Put bluntly, the humoural physiology of the eighteenth century is not the same as the one mapped by Victorian anatomists or, indeed, by twenty-first-century neuroscientists. This is not a denial that brain activity, for instance, in all humans involves complex interactions between receptors, ion channels, nucleic acids, and enzymes. But those interactions only make sense in social and environmental contexts. The question becomes: if a society does not have a concept of the circulation of the blood (as in the seventeenth century), does blood circulate? Yes, but not as we know or – importantly – experience it. Obviously, blood is doing something: it is moving according to the heavenly planets, for instance: but that is an entirely different thing. Crucially, the *choice* of figurative language tells its own, covert tale about underlining physiological beliefs. Physiological models of the body draw attention to certain things and not others, fundamentally affecting what is *noticed* – that is, *and* given meaning – and what is regarded as incidental. The physiological body is constituted by the figurative languages that bring the body into the world. Figurative languages ‘disclose’ our being-in-the-world.

## 7. Humoural physiology

This point can be illustrated by returning to the conceptual metaphors discussed by the cognitive linguists earlier in this article. It turns out that the physiological body upon which their schemata are drawn rests upon a historically specific, Western conception of physiology.

But what if people in the past conceptualized physiological ‘facts’ in completely different ways? The most obvious set of metaphors that people in past centuries drew upon to constitute the physiological body emerged from humoural theory, which was dominant for much of the period before the nineteenth century. The humoural body consisted of four fluids – phlegm, black bile, yellow bile, and blood. Linked to these humours were personality types (sanguine and melancholic). There were also three kinds of spirits, which acted on the humours: the natural, the vital, and the animal. In this model – unlike the biomedical one that was dominant until the 1960s – distinctions between bodies, minds, and souls were not clear-cut. Pain was the result of disequilibrium or imbalance. Illness was the result of disrupted relationships as much as disrupted physiologies. In the words of historian Ulinka Rublack, writing about a sixteenth-century ambassador (Bushecq) who fell ill:

The body itself was not regarded as a whole and clearly delimited entity, but rather … was understood as something that was constantly changing, absorbing and excreting, flowing, sweating, being bled, cupped and purged. It was clearly situated in the continually-changing context of a relationship to the world whose precise effect was never stable or predictable, so that one simply had to submit to it – to the terror that froze the blood, the sudden trembling, bleeding, or urination that literally stopped the ambassador Bushecq in his tracks. (2002, 2)

As a result, humoural theory provided rich figurative languages of ebbs and flows. To illustrate the vast differences between the ‘natural’ physiological processes described by the cognitive linguists in the late twentieth and early twenty-first centuries, and that of the eighteenth century and earlier, take Hervey's 1731 description of his sister's suffering. She was:

choked with phlegm, tormented with a constant cough, perpetual sickness at her stomach, most acute pains in her limbs, hysterical fits, knotted swellings about her neck and in her joints, and all sorts of disorders, consequent to a vitiated viscid [*sic*] blood, which, too glutinous and weak to perform its proper circulation, stops at every narrow passage in its progress, causes exquisite pains in all the little, irritated, distended vessels of the body, produces tumours in those that stretch most easily, and keeps the stomach and bowels constantly clogged, griped, and labouring, by the perspirable matter reverting there for want of force to make its due secretions and evacuate itself through its natural channels in the habit and the pores of the skin. ([1731] 1931, 971)

Pain in this account is a blockage of natural flows. It pervades all parts of the body, and not just particular organs.

This physiology is difficult to reconcile with that assumed by the current cognitive linguists. Of course, the most basic schema survive: ones based on sensorimotor bodily experience of lying down and standing up (happy is up and sad is down), for instance, or those based on physical comportment relating to vigour versus infirmity (health/life are up and illness/death are down). But others take on such a different meaning in the humoural scheme of physiology to be radically different. Take the very prominent image schema balance, as in the metaphor ‘pain weighed down her spirits’. Earlier, I quoted Johnson as saying that balancing ‘is an activity we learn with our bodies and not by grasping a set of rules or concepts’ (1990, 74). According to Strathern, it is related to ‘equilibrium in the body, whereby we try to maintain an even state’ (1996, 183). The problem is that the notion of ‘equilibrium in the body’ is a central tenet of humoural thinking, and one that meant something very different in the eighteenth century in comparison to its meaning for Johnson and Strathern in the late twentieth century. For eighteenth-century commentators, balance in the physiological body referred as much to the flow of animal spirits (within and between persons), the alignment of the planets, interpersonal relations, diet, and the weather as it did to equilibrium linked to poise and steadiness of a body in the environment.

A similar point can be made about the two conceptual metaphors intensity is heat and anger is a heated fluid in a container. These metaphors, we are told, arise from the physiological fact that ‘vigorous bodily activity produces an increase in bodily heat’; it is the way our body automatically responds, thus producing the conceptual metaphor: intensity is heat (Kövecses 2005, 18). But what if that person chopping firewood or vigorously making love understands the process of getting hot under the collar (or sheets) not so much in terms of thermoregulation controlled by neurons in the brain's hypothalamus, but in terms of the excretion of excess humours? This different physiological understanding leads as much to equilibrium is heat or excreting surpluses is heat, as to intensity is heat. Indeed, it might even lead to the conceptual metaphor moderation is heat, in the sense that returning the body to its correct, temperate level creates heat. Of course, this is not to deny that in both centuries people will get hot under the collar or sheets when working or making love, but to claim that this informs a universal physiology which is then mapped onto universal, conceptual metaphors does not tell us much about the experience of different bodies.

Let me just give a few examples of conceptual metaphors employed in the humoural body that would make little sense in the biomedical body. The chief one is pain is movement. As scholar Thomas Gray described his pains in 1755, they ‘wandering’ throughout his ‘constitution’, until they ‘fix[ed] into the Gout’ (1755, np). Pain ‘rolls along sluggishly or like a Wool pack’, in the words of pauper Mary Brooks at Guy's Hospital in November 1810 (Anon. 1810, np). Pain in these accounts was a blockage of natural flows. It pervaded all parts of the body, and not just particular organs.

In this physiological mapping, pain circulates: chased out of one part, it migrated to another. As Young described it in 1762:

I have been troubled near thirty years, with Rheumatic *Pains*; they have been now long entirely ceased; and my Physitians [*sic*] tell me, that [now] Nature throws all that Mischief on my Eyes, & Head. (np)

Walpole, writing in 1765, was ‘seized with the gout in one foot at the End of June, soon had it in both, with great torment, & then without its going out of my feet, in head, Stomach, both wrists & both Shoulders’ (1765, np). This was also the language used by ‘E.C.’ a patient in the London Dispensary, who described in 1811 ‘a pain in the Stomach, which flew to her head; the pain seemed at first … more like a stagnation’ (Rees 1811, 63).

Given such ways of understanding the body, it made little sense to distinguish physical from mental pain. An individual's temperament, what she ate or drank, the climate, and relationships with other people all affected her pain. Thus, we get the conceptual metaphor pain is a stagnating environment, as in Atkins' description of pain as a ‘slow stagnated fever … extreme pain in his side, breast, and bowels &c.’ ([1781] 1975, 55). pain is changing temperature (from the humoural physiology) and (in the solidistic tradition of eighteenth-century Scottish physicians like William Cullen) pain is inelasticity are two other important schemata in this conception of the body. The Hot/Cold and Wet/Dry basis of humoural medicine created major anxieties about change in temperature. Indeed, it was the most prominent language for pain in eighteenth-century narratives. In the words of a Bristol carpenter, he was ‘at a house at work in Clifton, it was very hot, and I drank some cold water, then I was laid up for a week with a bad Stummick’ (J. Bennett, Manuscript Autobiography in the Bristol Record Office, 8). Inelasticity appears almost as frequently, as in Cheyne's description of pain as the result of having ‘filled the original lax Membranes and Vessels [too] full, and they being somewhat broken are not sufficiently strong and elastic to force out the perspireable Wind and Steams which being retained perpetrate on the Membranes’ ([1733–43] 1943, 61–62).

My point is *not* that we should jettison the idea that the physiological body has a significant influence on the way people think. Rather, that the body which is conceived of in terms of autonomic arousal, cardiovascular responses, and sensorimotor actions is dependent on a very modern conception of the physiological body. Like the cognitive linguists, I agree that an analysis of conceptual metaphors illustrates some of the ways in which people *think* via physiological experiences: that is, our minds are embodied and the body is ‘mind-ful’. My point is that twentieth- and twenty-first-century theorists project a very modern conception of what constitutes that physiology, as though it is the ‘natural’ one. The physiological body might be so subtly dissimilar in the past to the modern one, to render processes of ‘embodiment’ and ‘thinking via physiological experiences’ profoundly different. Gibbs rightly quipped that ‘cognition is what happens when the body meets the world’: we should just not assume that what ‘happens’ is happening to the same body, and that cognitive responses are therefore identical (1999, 153).

As I have argued in the last two sections, figurative languages are important in constituting the physiological body. I used the example of the figurative languages of humoural physiology to argue that eighteenth-century bodies-in-pain *felt* different to modern ones. The figurative languages of humoural bodies reveal different ways of being-in-the-world.

It is important to observe, though, that causality could also go the other direction: physiological ‘facts’ (as understood in the context of the times) could also turn into metaphorical commonplaces. Indeed, this is precisely what happened with the physiology of the sympathetic nervous system, which was invented in the mid-eighteenth century as a *physiological* principle (a ‘sentient principle’) (for example, see Jackson [1781, 13]; Ord [1836, 26–27]; Whytt [1768, 583]) and only later in the early nineteenth century was turned metaphorically into a *social* system (sympathizing with others) (see Bourke 2012).

## 8. Embodiment and social context

Up to this point in this article, I have explored interactions between the body and metaphor. I have addressed the problem that such individual-based processes (particularly those processes dependent upon physiological activity) wrongly imply that the body and, therefore, metaphors are universal. Although I focused primarily on dialogues between the body and metaphor, when I turned to critique assumptions about the universality of human physiology, I was required to pay attention to the effect of social and environmental interactions on not only *representing* the body, but also in *creating* it. The following section develops this argument, emphasizing the effect of interpersonal and environmental interactions. In other words, so far, I have been principally concerned with two strands in my model: metaphor and body. Now, however, I turn to the third strand: cultural interaction. The body that creates language and metaphor is a social entity. The entwining of body and language only occurs within social contexts. In the words of philosopher Ludwig Wittgenstein, ‘mental language is rendered significant not by virtue of its capacity to reveal, mark, or describe mental states, but by its function in social interaction’ (1953, 188). Sensations of pain arise in the context of complex interactions within the environment, including interactions with other people.

Bodily sensations and its metaphors do not emerge fully formed out of an individual's head but are forged in interaction with other social worlds from infancy onwards: metaphor-creation is a social phenomenon. This is one of the contributions anthropologist Thomas Csordas has made to the debates. He began with the familiar statement that the image schema for containment is ‘based on one's own bodily experience of things going in and out of the body, and of our body going in and out of containers’. However, he rightly observes, containment is much more than simply a ‘sensori-motor act’: it was sometimes:

an event full of anticipation, sometimes surprise, sometimes fear, sometimes joy, each of which are shaped by the presence of other objects and people that we interact with. Image schemas are not therefore simply given by the body but reconstructed out of culturally governed interactions. (cited in Gibbs 1999, 154)

In other words, people choose their metaphors not as ‘contained’, isolated, individual bodies, but in interaction with other bodies and social environments. In the context of pain, it makes a difference whether pain was conceived of as being inflicted by an infuriated deity, due to imbalance in the ebb and flow of humours, emerged after a lifetime of ‘bad habits’, or resulted from an invasion by a germ.

Pain-metaphors can also arise out of interactions within the environment, including interactions with other people. As I have argued elsewhere, there is no necessary and proportionate connection between the intensity of tissue damage and the amount of suffering experienced since phenomenon as different as battle enthusiasm, work satisfaction, spousal relationships, and the colour of the analgesic pill can determine the degree of pain felt (Bourke 2013, 2014). The profound ways that the body-in-pain is influenced by the environment and social interactions is at the heart of debates about phenomena as diverse as mesmerism, placebos, psychosomatic disorders, and so on.

Most frequently, pain-metaphors were drawn from everyday encounters – interactions with squalling infants, sticking plasters, over-the-counter medicines, pincushions, and household objects. For example, in the 1830s and 1840s, a man suffering toothache described himself as ‘swaying my body to and fro, as if endeavouring to calm a fractious infant’ (in both Anon. [1837, 21] and Anon. [1842, 16]). It was as if a ‘very strong sticking-plaster were dragging the flesh down the bone’, complained 43-year-old Hannah D. at the Royal Free Hospital in the 1890s (Head 1894, 375). Around the same time, another patient – this time at The London Hospital – was heard describing her pain thus:

Oh sister, I've got such a dreadful effervescing headache, and I took a Seidlitz powder, and it fizzed up and made it worse, and now the powder's settled behind my eyes and it's something awful. (Anon. 1899b, 193)

Clearly, metaphors drawn from everyday encounters with material objects (such as Seidlitz powder) were highly volatile: they emerged from the changing worlds of business and advertising. There were other, even starker, ways in which changes in environment resulted in dramatic shifts in the concrete images available to move a subject (in this case, pain) from inchoateness to concreteness. It is no coincidence, for instance, that the fascination associated with railways in the mid-nineteenth century entered the metaphoric languages of pain almost immediately: after all, railways lent themselves particularly well to the imagery of circulatory systems, nerves, and veins, with railway tracks as steel arteries; railway engines, throbbing inflammations. Pain could easily be depicted as a railway accident. In 1860s Britain, in particular, railway accidents inspired a series of panics, resulting not only in widely reported mass deaths but also in the invention of entirely new diagnostic categories, such as ‘railway spine’ (the predecessor for psychological trauma as understood in contemporary parlance). Pain-narratives rapidly transferred the concrete image of a railway accident into a completely different context – that of nerve-pain, for instance. In the words of physician Valentine Mott, writing in 1862, about the pain of neuralgia:

I have seen the most heroic and stout-hearted men shed tears like a child, when enduring the agony of neuralgia. As in a powerful engine when the director turns some little key, and the monster is at once aroused, and plunges along the pathway, screaming and breathing forth flames in the majesty of his power, so the hero of a hundred battles, if perchance a filament of nerve is compressed, is seized with spasms, and struggles to escape the unendurable agony. (5)

Mott drew on the masculine imagery of industry and war. For him, pain was a mechanical monster, reducing war heroes to children. It was a scream, like a train horn. It was the searing heat of stoked engines. As in railway accidents, it bore down upon a person by random (fixing on any particular individual by chance), and the cause of the disaster might be simple and small – nothing more than the compression of a ‘filament of nerve’ – but it was all-powerful and inescapable.

Railway engines and accidents were one of many tropes of the industrial age that were translated into the context of physical pain. Typically, the distressed body was spoken about as if it were a flawed machine, with the physician as a kind of mechanic whose job it was to ‘fix’ the mechanism. As one patient puts it in the 1960s, pain was caused by ‘rust around the nerves’, ‘defective ball bearings’, or ‘twisted ligaments’ (Zborowski 1969, 85). Not surprisingly, mechanical metaphors – with their association with masculine occupations – were more likely to be the way men (rather than women) communicated painful sensations. Men even drew on personal experiences of mechanical engineering. In the words of one such man describing nerve-pain in the 1960s:

That's – that's my nerve – that's very vital. Nerves is a vital thing. I'm not a dummy – I can understand, you know, very well. I know how to fix an automobile, and if you know how to fix it right you got to be smart, you can't be a dummy. I know that nerves are vital. You can cut a nerve – that's the end of the nerve. You cut your leg off and you get a wooden one. But you can't get a nerve. (Zborowski 1969, 87)

Like a broken-down car, spare parts could be found for certain parts of the body – limbs, for instance – other parts, such as nerves, were irreplaceable.

Electricity was another technology that rapidly entered into languages of pain. It was widely employed in pain-metaphors from early nineteenth century. It may have been a particularly apt metaphor to convey the sensation of pain – and not only because of its properties as attacking unexpectedly and with dramatic power (related, of course, to lightning). In addition, though, the metaphorical link between pain and electricity may also have been related to the fact that it had begun to play an important role as a therapeutic agent against pain. From the 1850s, for example, the mass-produced Pulvermacher promised to ‘speedily sooth[e] agonizing pains’ with an electrical current (for example, see Anon. [1856, 108]; Anon. [1870, 642]). It is not surprising, then, that electrical metaphors became increasingly common in pain-discourses. In 1878, for instance, a man described his pain ‘like electric shocks in both legs’ (Buzzard 1878, 181). ‘The Young Lord's Adventure’ (1886) included a passage where every time the hero touched ‘one of the tiny men’, a ‘sharp, tingling pain, like a powerful shock of electricity’ shot through his body (Leys 1886, 244). In 1893, neuralgia was described as a form of ‘excruciating agony’ that might ‘appear with the suddenness of an electric shock’ (Anon. 1893, ix). In the 1930s, a 50-year-old woman described ‘burning pains in the left upper limb’ like ‘radiating shocks of electricity’ (Leriche 1939, 120). As one patient suffering Trigeminal neuralgia puts it in the 1960s, ‘My pain was caused by a short of two nerves – it's like electricity. If you put two nerves together and they touch each other, it forms a short and that's why I got my pain’ (Zborowski 1969, 85). In this way, metaphors reflected tangible changes in the material environment, which could be adopted to help describe inchoate sensations.

War metaphors were also prominent. Pain ‘cracked like the firing of a pistol’ (Townend 1869, 18). A ‘mighty pain as if a lyddite shell had hit’ overwhelmed a wrestler, according to one account of 1900, just four years after the introduction of that explosive into the British army (Anon. 1900, 7). Trigeminal neuralgic was described as coming ‘in a succession of short, sharp momentary bursts like electric shocks or machine-gun fire’ (Miller 1968, 577). Furthermore, although war metaphors had been used since the Middle Ages to describe pain (Montgomery 1991, 341–391), they became increasingly prominent in pain-talk during times of conflict.

The increase in militaristic metaphors in pain-talk was only partly a result of the militarization of society in the context of the two world wars and then the threats contained in nuclear technologies. It may also have been a response to the introduction of more effective analgesics, such as aspirin. After all, these medical technologies were aggressively marketed in militaristic terms. For instance, the first use in *The Times* of the term ‘kill’ in medical advertisements for pain-relief occurred in 1941 with the headline ‘Genaspirin Kills Pain Quickly – Time It!’ (Anon. 1941), in which a female office worker claimed that she could not ‘waste time having headaches now that we're short-staffed’ so she took two Genaspirin tablets: her pain was quickly ‘killed’. It was during the Second World War that cancer was also described for the first time in these advertisements in militarist terms. ‘Defeat the Silent Enemy’ (Anon. 1940), declared the advertisement in 1940: donations were required for the Royal Cancer Hospital in order to ‘swiften [*sic*] the attack on Cancer wherever it raises its hideous head … Cancer attacks without declaring War’ (7). Pain was no longer conceived of as an entity that had to be passively endured. Rather, it was an ‘enemy’ to be fought and ultimately defeated. When the pharmaceutical possibility of eradicating acute and chronic pain was limited, endurance could be valourized as a virtue: the introduction of effective relief (at least for acute pain) made passive endurance perverse rather than praiseworthy. In the latter case, it was the duty of both patient and physician to tackle the problem of pain, all guns blazing.

While industrial, mechanical, and militaristic metaphors were multiplying, others underwent a slow decline. Sometimes this can be explained in educational terms: with the decline of a classical education, including Latin and Greek, metaphors drawn from the classics evaporated. It would be rare, for instance, to hear anyone today refer to bodily agony in terms of the bronze bull, made for Phalaris (the tyrant of Acragas in Sicily), in which he would roast his enemies alive. But this was the way Swift (author of *Gulliver's Travels*) described his gout in 1740. ‘I am and have been these two days in so miserable a way, and so cruelly tortured, that can hardly be conceived’, he grumbled to his cousin Martha Whiteway, adding that the ‘whole last night I was equally struck as if I had been in Phalaris's brazen bull and roared as loud for eight or nine hours’ (np).

Similarly, although it was common throughout the period to refer to pain as torture (as in an 1862 description of ‘those horrible rhumatic [*sic*] tortures’) (Moodie [1862] 1985, np; also see, Anon. [n.d., np]; Waggett [1936, 1036]), in periods when torture was a judicial reality, torture metaphors were not only more common but were also more elaborate. So, in 1756 when Gray described John Chute as having experienced ‘the Gout for these five days with such a degree of pain & uneasiness, as he never felt before’, he also reported that, for 40 hours, ‘it seem'd past all human suffering, & he lay screaming like a Man upon the rack. [*sic*] the torture was so great’ (np). ‘Nature’ and rural metaphors for pain also declined. Pain that resembled ‘dogs … biting him’ (Sidless 1778, np) could be heard throughout the period, but less frequently in increasingly urbanized environments where dogs were more likely to be pampered pets than work-dogs or strays. Similarly, pain that ‘flickered’ like candles or oil lamps was more common when these were the dominant form of lighting. This is one reason why a comment by Rees in 1811 is intriguing. Rees was referring to a 35-year-old patient called Mrs W. who ‘complains of great weakness and internal sinking … violent Spasms at times, which almost stop her respiration, and shoot from the pit of the Stomach’. He noted that her symptoms included ‘a flickering at the stomach’. After the word ‘flickering’, however, Rees inserted a footnote: the word ‘flickering’, he commented, was ‘frequently made use of by the common people’. It was ‘a kind of onomatopoeia which is easily understood, [so] I have used it, that the case may be conveyed as far as possible in the language of the patient’ (1811, 191). It was a pain-metaphor that would decline with the advent of electric light bulbs that ‘shocked’, ‘sparked’, and ‘blew’ rather than flickered or spluttered.

However, the two largest groups of metaphors that underwent catastrophic decline were those of humours and religion. I have already mentioned the first of these shifts in the context of the very different understanding of the body according to humoural medicine. The fading away of humoural physiology was responsible for the increase in more individualized images of bodily pain: the body was more contained, more isolated. With germ theory, pain-metaphors became something much more mechanistic and invasive.

The decline in religious metaphors is also important, however. In the earlier period, pain was much more likely to be characterized as a demon or fiend; it propelled sufferers into hellfire. In 1816, a hypochondriac felt like he had ‘seven devils in my belly’, which could be cured by electric shocks from ‘a kind of machine’ (Anon. 1816, 144). In 1818:

That devil, call'd the Tooth-ache, comes,
Without an invitation;
And got tight hold of my stumps and gums,
And swore he'd keep his station. (Hudson 1818, 20)

In ‘The Toothache’ (Anon. 1833), the tooth ‘continued to ache, ache, ache, as if some fiend were beating and beating upon the nerve with his invisible and tormenting hammer … the fiend still beating and beating and beating with unrelenting perseverance’ (37). For a farm labourer in 1878, phantom limb pain (the result of his fingers being torn off by a ‘machine at Farmer Robinson's’) not only drove him ‘mad with an empty belly’ but also caused him ‘pain like hell-fire where your fingers ought to be’ (Anon. 1878, 71). In 1881, *The Sporting Times* even characterized the pain of neuralgia as a ‘demon’ that a sufferer could ‘drive away’ by a ‘nourishing, plentiful, and wholesome diet’ (which he described as including ‘plenty of good soups, oysters, rump steaks, &c. washed down with good stout or port wine, *not spirits*’) (Tupman 1881, 902).

The decline of religious metaphors also resulted in a reduction in the number of *positive* images of pain – bodily agony being an angel, for instance. According to these metaphors of pain, pain was God's sentinel. In the words of the author of *Cheering Views of Man and Providence*, ‘Who can calculate the self-destruction that would ensure, were it not for this vigilant sentinel, this stern commandment stationed in the frail body by Providence?’ (Burton 1832, 28–29). As another author concluded in 1854, pain was a ‘prayer uttered by the nerve for healthy blood’: it was ‘placed by our Maker as the beneficent guardian of this mortal fabric, a warning friend more often than an avenging angel’ (Sieveking 1854, 157). As the short story entitled ‘The Angel’ contended a few years later, pain was an angel ‘warning you of danger’. It was an angelic reminder that ‘imprudence’ (in this case, dressing ‘too thinly’ at night) would ‘bring its own punishment’ (Arthur [1858, 183]; also see Scholastica [1915, 150]; ‘W.G.W.’ [1918, 84]). The negative metaphors for pain (as hellfire or demonic) declined before those of pain as an angelic visitation, although both can still be found, but only in literature that is explicitly theological (Agnew and Mersky 1976, 73).

## 9. Conclusion

The relationship between body, language, and cultural interactions is a dynamic, inter-reactive one. Concepts within embodied cognition are useful to the historian because they provide a way of mapping the interactive nature of body and language *as it changes through time*, allowing us to speculate on historical shifts in sensation. Metaphors arise from the nature of our bodies, which, in turn, are in dialogue with metaphor. Bodies are not pure ‘soma’, but are constituted by social interactions and linguistic processes. Sensory perceptions are crucial in generating knowledge. Social environments and physiology map themselves strongly in the figurative languages people employ to communicate their pain. Cultural forces impose their own logic upon bodies and pain-narratives

This way of thinking about pain and the way it has been communicated in the past usefully muddies mind/body dualism. Its dynamic structure allows for the possibility of investigating *different* bodies (male, female, pink, brown, black, petite, obese, and so on), and, crucially for my project, it opens a space for exploring the ways in which painful sensations change over time. People's experiences of their bodies are shaped by environmental contexts and cultural processes, including language and dialect, power relations, gender, class and cultural expectations, and the weight and meaning given to religious, scientific, and other knowledges. Bodies are not simply entities awaiting social inscription (as implied in the ‘body-as-text’ metaphor), but are active agents in both creating social worlds and, in turn, being created by them. Human experience, in the words of Kirmayer, ‘emerges from our bodily being-in-the-world’. As Kirmayer points out, people's experiences:

reflect both the physiological machinery of the body and its cultural shaping through ongoing interaction with others across the lifespan. Physiology underwrites the stories that constitute the self, even as our self-depiction remodels bodily structures and reconfigures their functions. (2007, 363)

People are born into worlds that are not of their own making: they must navigate within this world, and they do so by employing not only the existing metaphorical tools but also the ability to imaginatively create other conceptual domains from bodily experiences. These metaphors do not merely reflect pain but are crucial in constituting it, within interactive, historical contexts.
